# Roles of Subunit ND2/NuoN in the Proton Pumping Coupling Mechanism of Complex I

**DOI:** 10.3390/ijms27072990

**Published:** 2026-03-25

**Authors:** Andrew E. Wadley, Madhavan Narayanan, Eiko Nakamaru-Ogiso

**Affiliations:** 1 Johnson Research Foundation, Department of Biochemistry and Biophysics, Perelman School of Medicine, University of Pennsylvania, Philadelphia, PA 19104, USA; 2 Mitochondrial Medicine Frontier Program, Division of Human Genetics, Department of Pediatrics, The Children’s Hospital of Philadelphia, Philadelphia, PA 19104, USA; 3 Department of Pediatrics, Perelman School of Medicine, University of Pennsylvania, PA 19104, USA

**Keywords:** CI, ND2, energy coupling, ubiquinone, proton pumping

## Abstract

Complex I (NADH:quinone oxidoreductase, CI) is central to cellular aerobic energy metabolism. The L-shaped structure of CI is unique, where the hydrophilic arm is responsible for the electron transfer function and the membrane arm operates proton pumping. These two functional sites are spatially far apart yet functionally connected. This basic core subunit architecture is highly conserved from bacterial to mammalian CI. Here, to gain detailed mechanistic insight into the role of the membrane subunit ND2 in the coupling mechanism, we mutated several highly conserved residues in the middle of the membrane axis of NuoN, the *E. coli* CI homolog of ND2. To more precisely investigate the consequences of mutational effects on highly conserved residues, we purified each mutant CI and compared the mutational effects on electron transfer and proton pumping activity using our instant membrane reconstitution method with *E. coli* double knockout (DKO) membrane vesicles lacking both CI and alternative NADH dehydrogenase (NDH-2). Thre results were corroborated by conventional proteoliposome reconstitution experiments. We found that Lys247 and Lys395 are absolutely essential for both electron transfer and proton pumping activities, while about 50% reduction of NADH oxidase activity but no reduction in proton pumping activity was observed in Lys217, and no significant decrease was detected in Glu133. Furthermore, unexpectedly, we were able to purify an NuoN knockout (ΔNuoN) mutant, which contained stoichiometric peripheral subunits NuoB, NuoCD, NuoE, NuoF, NuoG, and NuoI; and a substoichiometric amount of NuoH and a reduced amount of quinone. However, surprisingly, this isolated ΔNuoN CI showed CI activities (~30% of the WT) after being reconstituted into DKO membranes but not into proteoliposomes. Later, we confirmed by blue native PAGE that the wild-type CI was partially formed from ΔNuoN CI by recruiting its missing membrane subunits that existed in DKO membranes. Our data strongly suggest that ND2/NuoN plays an essential role in the coupling mechanism in CI. CI is the entry respiratory chain enzyme and is central to cellular energy metabolism. Two highly conserved lysine residues in the center of the antiporter-like membrane subunit ND2 are essential for the coupling mechanism between electron transfer and proton translocation.

## 1. Introduction

Complex I (CI) plays a central role in cellular aerobic energy metabolism. Genomic sequence data have revealed that homologues of CI exist in bacteria, archaea, chloroplasts and mitochondria [1]. However, CI is the least understood component of the Respiratory Chain (RC), and our knowledge of CI catalytic mechanisms remains very limited. In mitochondria, CI provides ~40% of the proton-motive force required for ATP synthesis [2]. CI dysfunction has been implicated in a variety of mitochondrial diseases, including heart failure, type 2 diabetes, and neurodegenerative diseases such as Parkinson’s disease [3]. Therefore, mechanistic studies of CI are imperative for understanding mitochondrial energy metabolism and human mitochondrial disease mechanisms.

CI is an L-shaped multi-subunit membrane protein complex that catalyzes the thermodynamically favorable transfer of two electrons from NADH to ubiquinone (UQ), while translocating protons across the membrane which generates a proton gradient essential for ATP synthesis [4,5,6,7]. Several X-ray crystal structures from bacterial and mitochondrial CI have shown [8,9,10] that flavin mononucleotide (FMN), a chain of seven Fe/S clusters [5], and UQ are localized in the hydrophilic peripheral domain, while proton pumping takes place in the hydrophobic membrane domain far from the catalytic site. The three membrane-bound transporter subunits ND2 (mitochondrial nomenclature)/NuoN (*E. coli* nomenclature), ND4/NuoM, and ND5/NuoL are similar to subunits of the bacterial Mrp (multiple resistance and pH adaptation) family of Na^+^/H^+^ antiporters [11,12]. It has been postulated that a long-range conformational change triggered by electron transfer from NADH to UQ activates proton pumping through these antiporter-like membrane subunits. However, the detailed mechanism of coupling remains unknown.

Several groups, including ours, have previously shown the functional importance of these three distal subunits ND2/NuoN, ND4/NuoM, and ND5/NuoL for the redox-linked proton pumping coupling mechanism through mutagenesis studies in *E. coli* CI [13,14,15,16,17]. Despite their location far from the redox center (at least 80–100 Å away), specific single site-missense mutations of highly conserved charged residues in the middle of the membrane domain could lead to a drastic reductions (2–20% of control) in both electron transfer and proton pumping activities. However, there was no effect on peripheral NADH:K_3_Fe(CN)_6_ reductase activities. This suggests that these key charged residues are vital for the energy coupling mechanism in CI.

To gain more detailed mechanistic insight, in this study, we focused on the NuoN (the ND2 homolog of *E. coli* CI) subunit. Sequence comparisons suggested that ND2/NuoN of CI bears a striking resemblance to MrpA and MrpD of Mrp antiporters [12]. MrpA (E140 and K223) and MrpD (E137A and K219A) in *B. pseudofirmus* are well conserved in NuoL (E144 and K229), NuoM (E144 and K234), and NuoN (E133 and K217) [18]. However, it has been reported that TMs 1–3 of ND2 are missing in CI from higher metazoans [11,19], interestingly enabling its interaction with N-methyl-d-aspartate receptors (NMDARs) and involving regulation via the non-receptor tyrosine kinase Src [20]. In addition, ND2 has been suggested to provide a possible secondary quinone (Q) binding site [21]. These reports suggest that ND2/NuoN is functionally different and more diverse than NuoL and NuoM, beyond its role in H^+^ translocation.

In this study, to compare mutational effects more precisely, we purified each NuoN mutant CI from *E. coli* instead of using mutant membrane vesicles, which may contain different amounts of assembled CI due to the mutation. In this way, we can clearly interpret lower CI activities as a consequence of the mutations. In order to purify each NuoN mutant, we generated each NuoN mutation in a strain (MC4100/His_9_*nuoE*) containing a nine histidine coding sequence inserted upstream of *nuoE* in the chromosome by homologous recombination using pKO3 vectors [22]. We generated seven mutants in conserved charged residues located in the central axis of NuoN in addition to an NuoN knockout mutant (ΔNuoN), and we also created a revertant wild type. We studied how these mutations affected the electron transfer and proton pumping activities of CI. Also, to measure the proton pumping activities of isolated CI in a convenient way, we applied a simple and quick reconstitution method using *E. coli* double knockout (DKO) membrane vesicles lacking both CI and an alternative NADH dehydrogenase (NDH-2), resulting in no detectable NADH oxidase and H^+^ pumping activities [23]. We confirmed those results with conventional proteoliposome experiments.

## 2. Results

### 2.1. Sequence Analysis of the ND2/NuoN Subunit and Generation of Site-Specific Mutations

**Figure 1:**
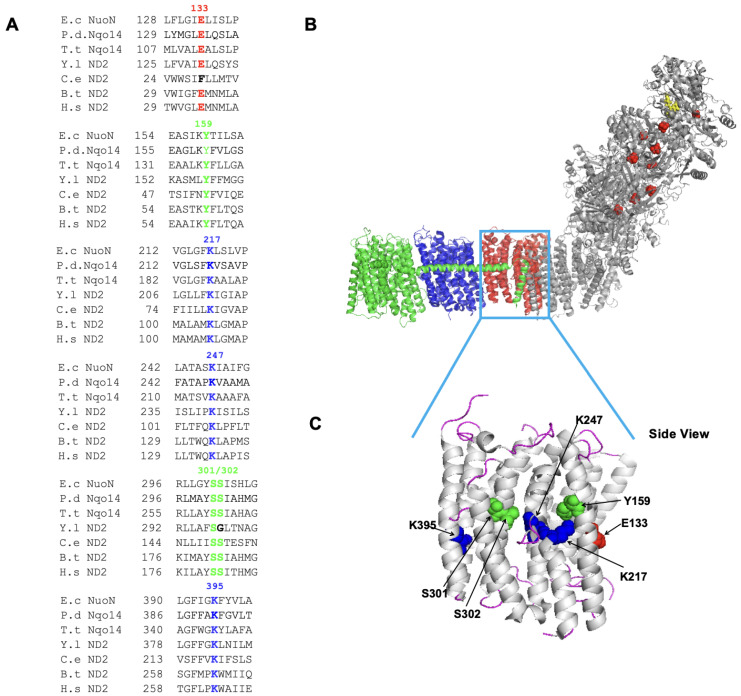
(**A**) Multiple sequence alignments of the mutated sites in the NuoN subunit of CI. GenBank Accession Numbers used are: E.c (*Escherichia coli*), AAC75336; P.d (*Paracoccus denitrificans*), AAA25600; T.t (*Thermus thermophilus*), AAA97951; Y.l (*Yarrowia lipolytica*), NP_075445; C.e (*Caenorhabditis elegans*), NP_006957; B.t (*Bos taurus*), AAQ06594; H.s (*Homo sapiens*), ADT80382. (**B**) Three-dimensional crystal structure of *T. thermophilus* CI (PDBID: 4HEA), showing the subunits NuoN, NuoM, and NuoL in red, blue, and green, respectively. (**C**) NuoN subunit of *E. coli* CI (PDB ID 3RKO). The locations of the mutated amino acids are shown in the NuoN subunit structure. The figure was generated using PyMOL (Version 1.8 (2015)).

We mutated 7 highly conserved residues—Glu133, Tyr159, Lys217, Lys247, Ser301, Ser302, and Lys395 (*E. coli* numbering)—in NuoN (Figure 1A). NuoN is localized to the middle of the membrane arm of CI (Figure 1B). The locations of these mutated amino acids are shown in Figure 1C. All the sites are in the central axis inside the membrane. We constructed these 7 site-directed NuoN point mutants in the (His)_9_-*nuoE* MC4100 strain [22] by chromosomal homologous recombination. All mutations were confirmed by DNA sequencing.

### 2.2. Purification of NuoN Mutant CI

**Figure 2:**
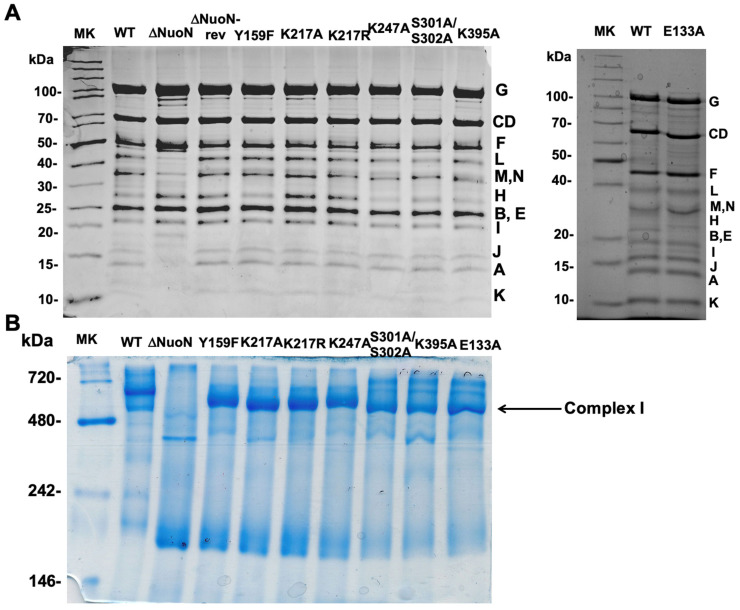
(**A**) SDS-PAGE of purified *E. coli* CI. Tricine SDS-PAGE on a 10% gel was performed according to [24]. *E. coli* CI consists of 13 different subunits: NuoA, NuoB, NuoCD, NuoE, NuoF, NuoG, NuoH, NuoI, NuoJ, NuoK, NuoL, NuoM, and NuoN. (**B**) Blue native PAGE [25] of purified CI WT and mutants. The arrow shows the location of fully assembled CI. Tris-Tricine SDS-PAGE gels and blue native gels were stained with Coomassie Brilliant Blue.

We generated and purified a total of 8 NuoN mutant CIs—E133A Y159F, K217A, K217R, K247A, S301A/S302A, K395A, ΔNuoN, and a NuoN revertant (a control NuoN mutant constructed with the unmutated gene pKO3 (NouN)—instead of pKO3 (nuoN mutants), in the recombination process) and the wild-type CI for comparison. Based on the SDS-PAGE patterns (Figure 2A), these purified NuoN CIs contain all the subunits and have similar purity. As shown in the blue native gel (Figure 2B), the sizes and amounts of assembled CI sizes are also comparable among all the mutants except ΔNuoN. We quantified quinone contents for ΔNuoN, K217A, K247A, and K395A, confirming that the bound Q amounts were close to those of the WT, except for ΔNuoN, in which most membrane subunits were missing (Table 1).

### 2.3. Proton Pumping and NADH Oxidase Activities of NuoN Mutant CI Using the DKO Instant Reconstitution Method

**Figure 3:**
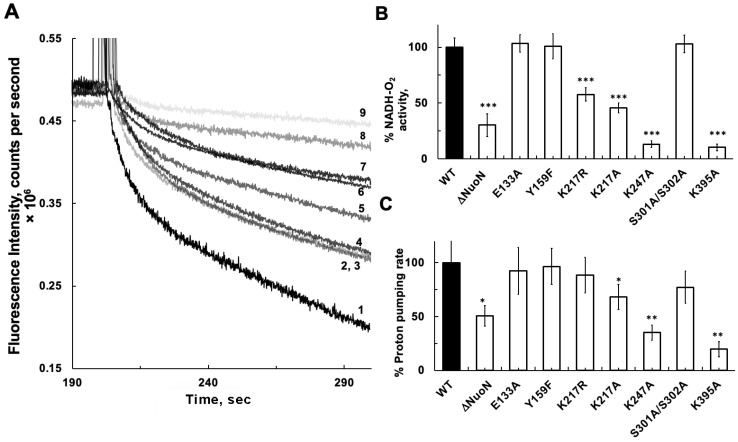
Proton pumping traces (**A**), NADH-oxidase activities (**B**), and proton pumping rates (**C**) of purified WT CI and the various NuoN mutants reconstituted in DKO membranes. The numbering in Figure 3 (**A**) represents: 1, WT; 2, S301A/S302A; 3, K217R; 4, Y159F; 5, K217A; 6, KO; 7, E133A; 8, K247A; 9, K395A. All measurements were carried out in a reaction mixture containing 50 mM MOPS/NaOH, pH 7.0, 50 mM KCl, and 20 mM MgCl_2_. ACMA (0.2 µM) was included in the proton pumping measurements. The reaction was initiated by adding NADH. Both NADH-oxidase and proton pumping activities were measured using the same reconstituted samples. Averages and standard deviations were calculated from 3–4 trials. Student’s *t*-test was performed between WT and each NuoN mutant: * *p* < 0.05, ** *p* < 0.01, and *** *p* < 0.001.

Preparing proteoliposomes requires a long processing time (~4 h) and a relatively large amount of protein. Therefore, we previously developed a simple and quick reconstitution method that requires only 5 min. We prepared membrane vesicles from DKO *E. coli* cells, which lacked both CI and NDH-2 [26]. The DKO membrane vesicles showed no NADH-oxidase activities or NADH-initiated proton pumping activities. Using ACMA as a delta-pH indicator, NADH-linked proton pumping activity in reconstituted NuoN mutant CI with the DKO membranes was measured after the addition of NADH. Representative traces of each NuoN mutant CI are shown in Figure 3A, and the averages of three measurements for NADH-oxidase and proton pumping activities are shown in Figure 3B and Figure 3C, respectively. Both NADH-oxidase activities and proton pumping were almost perfectly maintained in E133A and Y159F at the WT levels. NADH-oxidase activities in K217R and K217A became significantly lower, at 57.7% and 45.7% of the WT, while their proton pumping activities were less reduced, at 88.4 and 68.1% of the WT level, respectively. No reduction was observed in the NADH-oxidase activity in S301A/S302A, but its proton pumping activity was reduced to 77.2% of the WT level. Both NADH-oxidase and proton pumping activities were severely affected in K247A and K395A mutants, at 13.1% and 35.2%, and 10.2% and 19.8% of the WT levels, respectively. Mutations at conserved lysine sites in the central membrane axis of the NuoN subunit affected electron transfer (NADH-oxidase) activity more severely than proton pumping activities, even though NuoN is situated far from the CI electron transfer pathway and quinone binding site(s). Our results strongly suggest that Lys247 and Lys395 are involved in the CI energy-coupling mechanism. To our utter surprise, we found that NADH-oxidase and proton pumping activities in ΔNuoN were 30.4% and 50.1% of the WT, much higher than those in K247A and K395A, despite the absence of fully assembled CIs as analyzed by BN-PAGE (Figure 2B, lane 2).

### 2.4. Proton Pumping and NADH Oxidase Activities of NuoN Mutant CI Using Proteoliposomes

**Figure 4:**
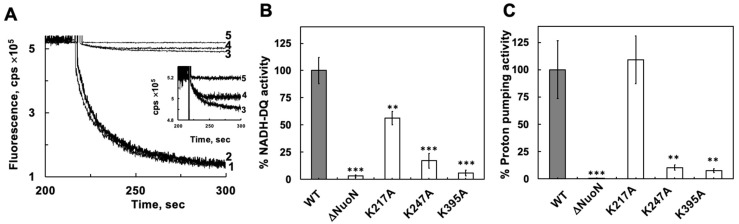
Proton pumping traces (**A**), NADH-DQ (**B**), and proton pumping activities (**C**) of purified WT CI and the various NuoN mutants reconstituted in *E. coli* polar lipid proteoliposomes. All measurements were carried out in a reaction mixture containing 5 mM MOPS/NaOH, pH 7.0, 50 mM KCl, and 400 µM MgCl_2_. A total of 0.2 µM ACMA was included in the proton pumping measurements. The reaction was initiated by the addition of NADH. Averages and standard deviations were calculated from 4 trials. Student’s *t*-test was performed between WT and each NuoN mutant: ** *p* < 0.01, and *** *p* < 0.001.

We also used the canonical proteoliposome reconstitution method to further confirm the mutational effects of the highly conserved lysine residues Lys217, Lys247, and Lys395 in the central membrane axis of NuoN. By this proteoliposome reconstitution method, the reduction of NADH-oxidase activity in K217A was confirmed, showing 56.4% of the WT (Figure 4B), a level similar to that measured by the DKO method (~50%) described above, while the proton pumping activity was normal (109%) with the proteoliposome method (Figure 4A,C), in contrast to ~70% of the WT by the DKO method. However, for the K247A and K395A mutants, severe CI functional defects were confirmed with the proteoliposome method; both NADH oxidase and proton pumping activities were severely reduced in K247A and K395A mutants, at 10.3% and 7.6%, and 17% and 5.7% of the WT levels, respectively. These results indicate that a purified CI is more tightly inserted into membranes by the conventional proteoliposomes, thus reflecting a more accurate proton pumping capacity compared to that of the CI inserted into DKO membranes by simple vortexing. However, the electron transfer activity values were very similar when measured by either the proteoliposome or DKO methods, probably because electron transfer from NADH to quinone requires a less rigid membrane environment. It is noteworthy to mention here that our simple reconstitution method using DKO membranes proved to be very useful for the initial screening to investigate functional phenotypes. Since both NADH-oxidase and proton pumping activities of CI require membranes, the presence of these activities in the reconstituted DKO membranes strongly suggests that CI was inserted in the right orientation. In fact, there was no difference in NADH:ferricyanide activities of these DKO membranes reconstituted with WT CI in the presence or absence of 1% DDM [23]. We can rule out the possibility that some population of CI was inserted in the wrong direction, which happens sometimes with proteoliposomes.

However, to our surprise, we did not see any detectable NADH-DQ or proton pumping activities in ΔNuoN, in striking contrast to the results from using the reconstituted DKO membranes. This proteoliposome result for ΔNuoN made more sense to us, as we did not see any assembled CI (Figure 2B) mentioned above.

**Figure 5:**
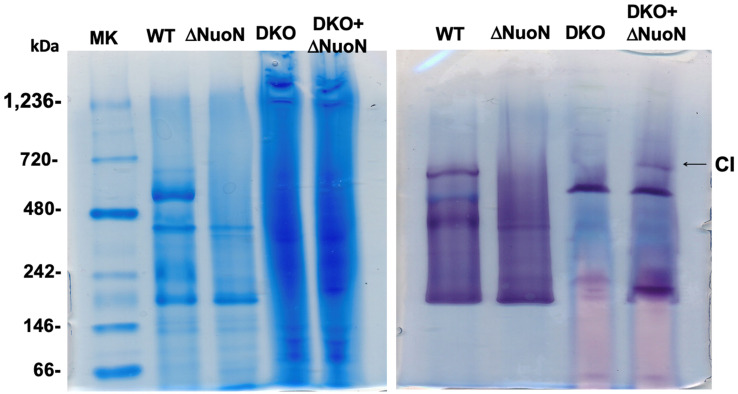
Blue native PAGE (**A**) and in-gel activity staining (**B**) of pure WT CI, KO, and reconstituted samples of WT and KO in DKO membranes. The arrow shows the location of fully assembled CI. Blue native gels were stained with Coomassie Brilliant Blue (**A**) or NBT (**B**), respectively.

To investigate why ΔNuoN showed significantly detectable activities of NADH-oxidase (~30% of the WT) and proton pumping (~50% of the WT) when it was reconstituted into the DKO membranes, BN-PAGE was performed for ΔNuoN CI reconstituted into DKO membranes and compared with purified WT CI, purified ΔNuoN CI, and DKO membranes. In-gel CI activity staining revealed that a fully assembled CI band surprisingly appeared in the reconstituted KO ΔNuoN, which does not exist in DKO membranes or purified ΔNuoN CI itself (Figure 5). This result suggests that upon reconstitution, purified ΔNuoN CI successfully recruited all the missing membrane subunits NuoA, NuoJ, NuoK, NuoL, NuoM, and NuoN, and probably more NuoH from DKO membranes, although NuoH was already seen in purified ΔNuoN CI but at a sub-stoichiometric level (Figure 2A, lane 2), resulting in the formation of a fully reassembled CI. Considering that the DKO membrane reconstitution procedure is so simple (just mixing by vortexing for 5 s), this phenomenon was beyond our imagination. This fact reasonably explains why ΔNuoN showed partial CI activities after its reconstitution into the DKO membranes.

**Figure 6:**
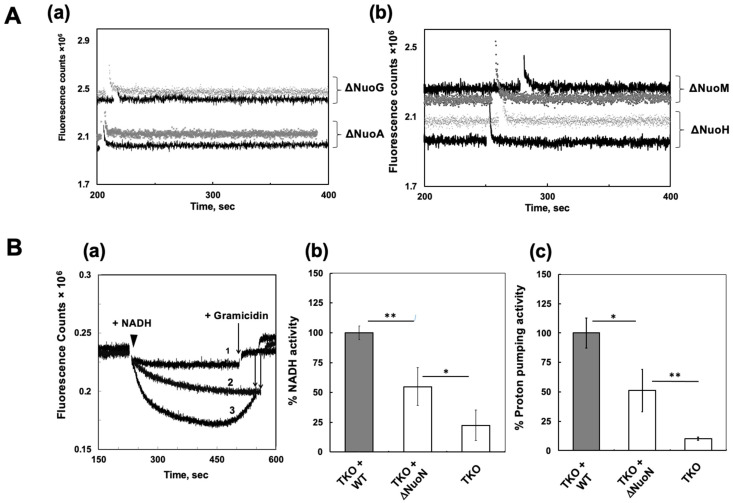
(**A**) Proton pumping traces of NuoA knockout (ΔNuoA) and NuoG knockout (ΔNuoG) membranes (**a**), as well as NuoM knockout (ΔNuoM) and NuoH knockout (ΔNuoH) membranes (**b**), with (dark lines) or without (light lines) ΔNuoN CI reconstitution. Proton pumping activities were initiated with dNADH, a selective substrate for CI. (**B**) (**a**) Proton pumping traces of TKO membrane (1), TKO membrane reconstituted with ΔNuoN CI (2), and TKO membrane reconstituted with WT CI (3). Proton pumping activities were initiated with NADH and terminated with gramicidin. Percentages of NADH-oxidase activities (**b**) and proton pumping rates (**c**) averaged from three technical replicates are shown. Student’s *t*-test was performed between TKO-WT and TKO-ΔNuoN or TKO-ΔNuoN and TKO: * *p* < 0.05 and ** *p* < 0.01.

We extended the instant reconstitution method to use other KO mutant membranes: ΔNuoA, ΔNuoG, ΔNuoH, and ΔNuoM. Since these KO membranes still contain NDH-2, we measured proton pumping activities initiated with diamino-NADH (a specific substrate for CI). None of the ΔNuoN CI reconstituted into those KO membranes showed any detectable proton pumping activities (Figure 6A). Western blot analysis against anti-NuoA and NuoL clearly showed that DKO membranes contain substantial amounts of subunits NuoA and NuoL, while ΔNuoA membranes lack the NuoL subunit, ΔNuoG and ΔNuoH membranes contain almost no NuoA and NuoL subunits, and ΔNuoM membranes contain both subunits at very low levels (Supplementary Figure S1) [27]. This clearly explains why no NADH-oxidase and proton pumping activities were found in those KO mutant membranes reconstituted with isolated ΔNuoN CI. To satisfy our curiosity, we also generated a TKO strain of *E. coli* devoid of cytochrome bo3, CI (a NuoB knockout), and NDH-2. We found that the TKO membranes contain both NuoA and NuoL subunits in amounts similar to those seen in DKO membranes (Supplementary Figure S1). And again, both NADH-oxidase and proton pumping activities were observed in these TKO membranes reconstituted with isolated ΔNuoN CI (Figure 6B). Since there is no complex III equivalent enzyme in *E. coli*, only CI and the essential terminal oxidase cytochrome bo3 carry out proton pumping in the *E. coli* respiration system. Therefore, importantly, our TKO results confirm that the proton pumping activity observed in TKO reconstituted with ΔNuoN CI was carried out by partially reassembled CI, and this result completely ruled out the possibility of proton pumping by cytochrome bo3, a downstream enzyme of CI.

## 3. Discussion

While impressive progress has been made in recent years in understanding the gigantic CI structure, elucidating the proton pumping mechanism of CI remains one of the most challenging bioenergetic problems. The structural complexity and the exceptionally high proton pumping stoichiometry of 4H^+^/2e^−^ (proton/electron) hamper our efforts to uncover the CI mechanism [28,29,30,31,32,33,34,35].

In this study, we investigated the functional roles of highly conserved amino acids situated in the central axis of ND2/NuoN, more than ~70 Å away from the quinone-reducing site [9]. To monitor proton pumping activities of isolated CI, purified proteins must be reconstituted into proteoliposomes. However, preparing proteoliposomes requires relatively large amounts of purified proteins and a long processing time (3–4 h). Therefore, we developed and employed a new, simple method for screening purposes. We first prepared *E. coli* DKO membrane vesicles containing no NADH oxidase and no NADH-initiated proton pumping activities. Mixing purified CI with DKO membranes by vigorous vortexing for 1 min was sufficient to analyze proton pumping activities. DKO membranes preserve the native *E. coli* lipid composition necessary for the stabilization and effective insertion of CI. We then validated the results with the conventional proteoliposome method. We mostly saw similar results with the two reconstitution methods. However, in the case of the K217A mutant, we observed a significant difference in proton pumping activity between the two methods (68% in DKO membranes versus 109% in proteoliposomes). We think that this difference is caused by the difference in the tightness of CI insertion into vesicles. We used the Bio-beads method for proteoliposome preparation: When beads were added to a detergent–protein–lipid mixture, the excess detergent molecules were efficiently adsorbed, facilitating formation of a tighter proteoliposome membrane. On the other hand, our instant DKO membrane reconstitution fully relies on the intrinsic association capacity driven by the membrane subunits only from the outside of the DKO membrane vesicles. Also, we observed a difference in the stability of formed vesicles: our reconstituted DKO membrane vesicles were only stable up to an hour or less on ice, while proteoliposomes were stable for at least several hours on ice.

In summary, as expected our data clearly confirmed the importance of highly conserved lysine residues. Lys247 and Lys395 are essential for both electron transfer and proton pumping activities, while Lys217 is important for electron transfer but not for proton pumping [15]. The lack of reduction in CI activities in E133A was also confirmed, suggesting that the role of Glu133 can be compensated by a nearby residue, the conserved Glu72 in NuoK [15,17]. The question we now have is why the electron transfer activity in K217A was reduced by ~50%, yet this mutant can still perform full proton pumping activity, indicating a gap between electron transfer and proton pumping function. A further study with this mutant is underway. In summary, our data with purified mutant CI clearly established the role of each Lys residue in CI function. Our results certainly suggest that ND2 plays an essential role in the coupling mechanism in CI via conserved lysine residues, acting as the main elements of the proton pumping mechanism.

Our major unexpected finding is that the purified ΔNuoN CI, containing all peripheral subunits (NuoB, NuoCD, NuoE, NuoF, NuoG, and NuoI) and a substoichiometric amount of NuoH, could recruit the other missing membrane subunits (NuoA, NuoJ, NuoK, NuoL, NuoM, and NuoN) existing in DKO membranes and reassemble a fully functional CI in vitro by simple mixing with DKO membranes. This phenomenon was also observed when purified ΔNuoN CI was mixed with TKO membranes, but not with other subunit KO membranes that lack the membrane subunits missing in ΔNuoN CI. These results can be seen as extreme examples in the molecular recognition field: Proteins are made to self-assemble spontaneously into multicomponent and complex structures, with instructions buried in their three-dimensional structure. However, we certainly admit the limitations of using DKO membranes when reconstituted with purified KO mutant CI missing certain membrane subunits. Therefore, we strongly suggest the need for confirmation experiments with proteoliposomes, although the phenomenon we observed with the purified ΔNuoN CI was an extremely rare event.

Human *MT-ND2* gene mutations could cause a broad spectrum of severe disorders like MELAS (mitochondrial encephalopathy, lactic acidosis, and stroke-like episodes) and Leigh syndrome, as well as milder disease phenotypes, including myopathies, migraine, dystonia, exercise intolerance, and lactic acidosis, also influencing metabolic health like type 2 diabetes. These diseases are all linked to energy production issues and increased oxidative stress [36]. Beyond its essential role in oxidative phosphorylation, *MT-ND2* genetic variation appears to influence the production of reactive oxygen species [37,38] and the proper assembly of CI [39]. Polymorphisms in *MT-ND2* have been linked to increased oxidative stress [40], which is thought to underlie heightened risks for cardiovascular events such as myocardial infarction and hypertension, as well as neurodegenerative and neuropathic conditions. Our findings reinforce the concept that ND2/NuoN is a key node integrating mitochondrial energy transduction with redox and cellular homeostasis.

In conclusion, our results strongly suggest that ND2/NuoN plays a central role in the proton pumping coupling mechanism in CI. More detailed studies of the role of ND2/NuoN are necessary to understand the CI coupling mechanisms and human *MT-ND2* disease.

## 4. Materials and Methods

### 4.1. Preparation of the Knock-Out NuoN (∆NuoN) and Mutant Strains in the (His)_9_-NuoE MC4100 Cells

The (His)_9_-*nuoE* MC4100 (*F*^−^, *araD139*, Δ(*arg F-lac*)*U169*, *ptsF25*, *relA1*, *flb5301*, *rpsL 150*.λ^−^) strain was generated previously for efficient purification purposes [22]. The ∆NuoN [Δ(*nuoN*::Spc)] and site-specific mutant NuoN strains were generated in this strain using the same method described previously [13]. The presence of mutations in the genomic gene was verified by PCR and DNA sequencing. The primers used to introduce a site-specific mutation into *E. coli* NuoN subunit are listed in Table 2.

### 4.2. Isolation of CI

*E. coli* CI was isolated from the wild-type (WT), ΔNuoN, and NuoN mutant strains by following the procedure published previously [22]. Briefly, CI was extracted from the membrane fraction with dodecyl-β-d-maltoside (DDM) at a final concentration of 1.2% (*w*/*v*), isolated using Ni-NTA resin, desalted, and concentrated to 3–8 mg protein/mL. The purified enzyme was quickly frozen in liquid nitrogen and stored at −80 °C until use.

### 4.3. Preparation of E. coli Double Knock-Out (DKO) Membrane Vesicles Lacking CI and NDH-2

We aerobically grew the DKO strain, *E. coli* ΔN1b/Δ*ndh* (DKO) [26], and prepared DKO membrane vesicles according to [41]. In brief, the cells were grown in 250 mL of Terrific Broth medium until an A_600_ of ~6 was reached and then harvested at 5800× *g* for 10 min. The cells were resuspended at 10% (*w*/*v*) in a buffer containing 50 mM Bis-Tris (pH 6.0), 1 mM EDTA, 1 mM DTT, 1 mM PMSF, and 10% (*w*/*v*) glycerol. The cell suspensions were sonicated twice for 1 min, passed twice through a French press at 25,000 p.s.i. and centrifuged at 23,400× *g* for 10 min. The supernatant was ultracentrifuged at 256,600× *g* for 1 h. The pellet was resuspended in the same buffer as described above. The resulting membrane vesicles were frozen in liquid nitrogen and stored in small aliquots at −80 °C until use. Membrane vesicles from *E. coli* ΔNuoA [41], ΔNuoG [42], ΔNuoH [43], and ΔNuoM [44] were also prepared for reconstitution experiments with purified ΔNuoN CI using the same method.

### 4.4. Reconstitution of Purified CI into DKO Membrane

Purified WT and NuoN mutant CI were mixed with DKO membrane vesicles at a 1:20 ratio (typically, 0.5:10 mg/mL), centrifuged briefly and then placed on ice right before use. We measured proton pumping and NADH oxidase activities in parallel for each experiment.

### 4.5. Preparation of Proteoliposomes

Proteoliposomes were prepared using *E. coli* polar lipid (Avanti, Alabaster, AL, USA) according to ref. [45]. Briefly, the lipid was suspended in a 50 mM Bis-Tris buffer at pH 6.0 containing 50 mM NaCl at 8 mg/mL and sonicated after the addition of 2.5% DDM until the lipid solution became clear. Then, the chilled liposome solution was mixed with CI in a 4:1 ratio and incubated in a shaker for 5 min at 4 °C. Immediately, SM2-biobeads were added, and the sample mixture was shaken for 3 h at 4 °C. At the end of 3 h, the sample was washed to remove biobeads. The proteoliposomes were pelleted by ultracentrifugation and dissolved in 5 mM MOPS pH 7.0 containing 50 mM KCl.

### 4.6. Proton Translocation Activity

The generation of a proton gradient was determined by monitoring the fluorescence quenching of 9-amino-6-chloro-2-methoxyacridine (ACMA, Sigma, St. Louis, MO, USA). Between 2.5 and 5 μg of DKO-reconstituted membranes were added to the assay buffer containing 50 mM MOPS (pH 7.0), 50 mM KCl, 20 mM MgCl_2_ and 0.2 μM ACMA under standard conditions. The mixture was incubated at 30 °C for 3 min. Proton pumping activity of proteoliposomes was assessed under slightly different and optimized assay buffer conditions. Proteoliposomes (5–20 μL) were added to the assay buffer containing 5 mM MOPS (pH 7.0), 50 mM KCl, 30 μM decylubiquinone (DQ), and 0.5 μM ACMA and incubated at 30 °C for 3–5 min. The fluorescence was detected with a Fluomax-4 spectrofluorometer (Horiba, Piscataway, NJ, USA) at an excitation wavelength of 430 nm and an emission wavelength of 480 nm. The reaction was started by the addition of 150 μM NADH. The slopes for the initial acidification rates were calculated from the data using a 5 s time window starting 5 s after the addition of NADH.

### 4.7. Generation of Triple Knock-Out (TKO) E. coli Strains

We recently generated a TKO strain of *E. coli* devoid of cytochrome bo3, CI, and NDH-2. We purchased a double KO strain (WTZ15001: *F*^−^, Δ(*araD-araB*)*567*, Δ*lacZ4787*(*::rrnB-3*), Δ*cyoB788::kan*, *λ*^−^, Δ*nuoB869::FRT*, *rph-1*, Δ(*rhaD-rhaB*)*568*, *hsdR514*), which lacks cytochrome *bo3* and CI, from the Keio collection, and we subsequently knocked out the *ndh* gene (encoding *E. coli* NDH-2) in this strain using a gene replacement technique with pKO3 [46].

### 4.8. Other Analytical Procedures

NADH-oxidase and NADH:DQ activities were measured at 30 °C by adding 1–5 μg of DKO reconstituted membranes or CI-proteoliposomes to 5 mM MOPS buffer (pH 7.0) containing 50 mM KCl and 30 μM DQ (only for the NADH:DQ assay). After incubating at 30 °C for 3 min, the reaction was initiated by the addition of 150 μM NADH. The absorbance was measured at 340 nm using a Cary 60 spectrophotometer (Agilent, Santa Clara, CA, USA). The extinction coefficient of ϵ340 = 6.22 mM^−1^ cm^−1^ for NADH was used for activity calculations. Reported values are the averages of three to four measurements. The extraction and quantification of quinones in purified NuoN mutant CI were performed as described in ref. [22]. Protein estimation was routinely done using the method of Lowry et al. [47] and Bradford [48]. SDS-PAGE, Tricine SDS-Page, and blue native PAGE were carried out according to Laemmli [49], Schägger [24], and Schägger [25,50], respectively. Any variations from the procedures and other details are described in the figure legends. All measurements were technical replicates using the same lot of purified mutant CI isolated from pooled bacterial cell pellets of 3–6 culture 3 L flasks, separately grown in 800 mL of media per flask. Therefore, the purified mutant CI used for these assays already represents averaged biological variables unique to each mutant.

## Figures and Tables

**Table 1:** Quinone content of NuoN mutants (n = 3).

	WT	ΔNuoN	K217A	K247A	K395A
UQ8 μmoles per one mole of CI	1.99 ± 0.07 ^a^	0.5 ± 0.09	1.76 ± 0.03	1.46 ± 0.16	1.92 ± 0.16

^a^ This value was cited from Ref. [22].

**Table 2:** Primers for introduction of a site-specific mutation into *E. coli* NuoN subunit.

Mutation	Mutagenic Primer Sequence ^a^
E133A	5′-CTGTTCCTCGGTATCGCACTGATCTCTTTGCCGC-3′
Y159F	5′-GGAAGCCAGTATCAAATTCACCATCCTTTCTGCCGCAGCG-3′
K217A	5′-GGCCTCGGCTTCGCACTCTCTCTGGTGCCG-3′
K217R	5′-GGCCTCGGCTTCAGACTCTCTCTGGTGCCG-3′
K247A	5′-CCTGGCGACGGCGAGCGCAATCGCTATCTTCGGTGTGG-3′
S301A/S302A	5′-CGTCTGCTCGGTTACGCAGCTATCTCTCACCTCGGC-3′
K395A	5′-CGCTGGGCTTTATCGGTGCGTTCTACGTGCTGGCG-3′

^a^ Underline indicates mutation. All sequences correspond to forward primers.

## Data Availability

The original contributions presented in this study are included in the article/Supplementary Materials. Further inquiries can be directed to the corresponding author.

## Supplementary Materials

The following supporting information can be downloaded at https://www.mdpi.com/article/10.3390/ijms27072990/s1.

## Abbreviations

BN-PAGE, blue native polyacrylamide electrophoresis; DDM, dodecyl-β-d-maltoside; DKO, double knock-out; DQ, decylubiquinone; Mrp, multiple resistance and pH adaptation antiporter; NDH-2, NADH dehydrogenase type 2; Q, quinone; TKO, triple knock-out; UQ, ubiquinone.
