# Supplementary material for: Roles of Subunit ND2/NuoN in the Proton Pumping Coupling Mechanism of Complex I
Source: Int J Mol Sci. 2026 Mar 25;27(7):2990. doi: 10.3390/ijms27072990 (PMC13073174; doi:10.3390/ijms27072990)
Supplement: Supplementary file 1 [file ijms-27-02990-s001.zip › ijms-4135190-supplementary.pdf]

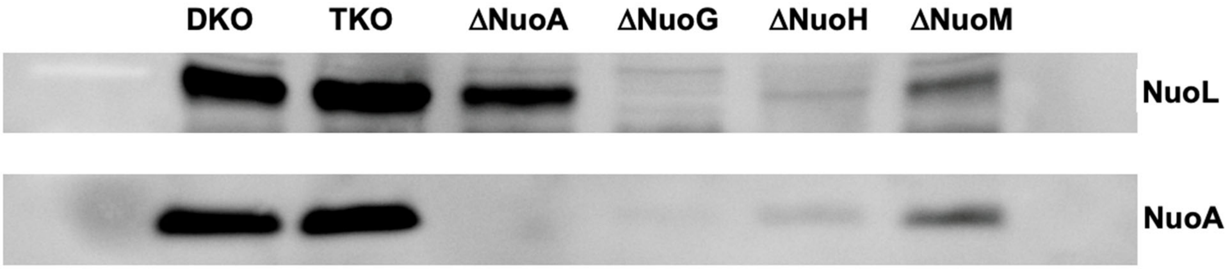

Supplemental Figure S1. Immunoblotting data performed with anti-NuoA and anti-NuoL antibodies generated in our lab [1, 2]. Western Blot samples are: 1, DKO; 2, TKO; 3 ΔNuoA; 4, ΔNuoG 5, ΔNuoH; 6, ΔNuoM. The presence of NuoL (black arrowhead) and NuoA (white arrowhead) was detected with antibodies. Immunoblotting was conducted as described previously [3].

1. Kao, M.C., et al., *Functional roles of four conserved charged residues in the membrane domain subunit NuoA of the proton-translocating NADH-quinone oxidoreductase from Escherichia coli*. J Biol Chem, 2004. **279**(31): p. 32360–6.
2. Nakamaru-Ogiso, E., et al., *The membrane subunit NuoL(ND5) is involved in the indirect proton pumping mechanism of Escherichia coli complex I*. J Biol Chem, 2010. **285**(50): p. 39070–8.
3. Nakamaru-Ogiso, E., et al., *Characterization of the iron-sulfur cluster N7 (N1c) in the subunit NuoG of the proton-translocating NADH-quinone oxidoreductase from Escherichia coli*. J Biol Chem, 2005. **280**(1): p. 301–7.
